# Changes in Digestive Health, Satiety and Overall Well-Being after 14 Days of a Multi-Functional GI Primer Supplement

**DOI:** 10.3390/nu16183173

**Published:** 2024-09-19

**Authors:** Elena Nekrasov, Alexandra Adorno Vita, Ryan Bradley, Nikhat Contractor, Nadeesha M. Gunaratne, Marissa Kuehn, Rick Kitisin, Deval Patel, Erin Woods, Bo Zhou

**Affiliations:** 1 Amway Innovation and Science, Ada, MI 49355, USA; 2 Helfgott Research Institute, National University of Natural Medicine, Portland, OR 97201, USA; 3 Amway Innovation and Science, Buena Park, CA 90621, USA; 4 Herbert Wertheim School of Public Health and Human Longevity Science, University of California, La Jolla, CA 92093, USA

**Keywords:** digestive health, well-being, supplements, prebiotics, postbiotics, digestive enzymes, gut microbiota, fermented foods, gut homeostasis, gut motility, functional food, dietary fiber

## Abstract

A recent review proposed a role for multi-functional food or supplement products in priming the gut to support both digestive and systemic health. Accordingly, we designed and eva-luated the effect of a multi-functional gastrointestinal (GI) primer supplement on participant-reported measures for digestive health, quality-of-life (e.g., energy/vitality and general health), and reasons for satiation (e.g., attitudes towards food and eating). In this single-arm clinical trial, 68 participants with mild digestive symptoms consumed the GI primer supplement daily for 14 days. Digestive symptoms were evaluated daily from baseline (Day 0) through Day 14. At baseline and Day 14, participants reported their stool consistency, reasons for satiation, and quality-of-life measures using validated questionnaires. At Day 14, participants reported significant improvements in all (13/13) digestive symptom parameters (*p*-values < 0.05) and an increase in % of stools with normal consistencies. There were significant improvements (*p*-values < 0.05) in energy/vitality and general health, and in specific attitudes towards food and eating (e.g., physical satisfaction, planned amount, decreased eating priority, decreased food appeal, and self-consciousness). Results suggest the GI primer supplement promotes digestive health, improves quality of life, and impacts attitudes towards food/eating. This study provides preliminary support for the gut priming hypothesis through which multi-functional digestive products may improve GI health.

## 1. Introduction

In recent years, gut mucosal health has established itself as a pivotal component of overall human health [1], playing a crucial role in nutrient absorption [2,3], cardiometabolic activities [4,5], immunoregulation [6,7], mental well-being [8,9], and more. Mucosal health can be impacted by many environmental exposures that travel through the digestive tract (e.g., food, xenobiotics, pathogens, etc.) and is maintained through the resilience of the gut microbiome and its interactions with gastrointestinal cell types (e.g., enterocytes, enteric immune cells, etc.). This concept of resilience—or the ability of gut microbiota and local cells to adequately respond to stressors and return the extracellular and intracellular environments/ecosystems to homeostatic conditions [10,11]—is key for maintaining not just local mucosal health but systemic health as well.

There are many mechanisms through which alterations in gut health lead to changes in local digestive health and systemic health. Insults to mucosal health can manifest as alterations in gut microbiota, disrupted digestive and absorptive processes, loss of barrier integrity, inflammatory activities, and the altered production of neurotransmitters and hormones in the gut. The digestive symptoms that may arise due to these changes (bloating, constipation, indigestion, etc.) can significantly affect one’s quality of life [12]. On the other hand, aside from physical discomfort and gastrointestinal pathology, these insults to mucosal health can subsequently lead to changes in systemic health. The gut–brain axis underscores how these alterations can affect mental health, influencing one’s mood, energy levels, cognitive functions, and emotional stability [9]. Inadequate nutrient absorption from an imbalanced digestive system may lead to suboptimal health and contribute to symptoms such as fatigue [12]. Moreover, poor intestinal barrier health and alterations in gut microbiota have been linked to a range of cardiometabolic and inflammatory diseases [1,4,13].

**Figure 1:**
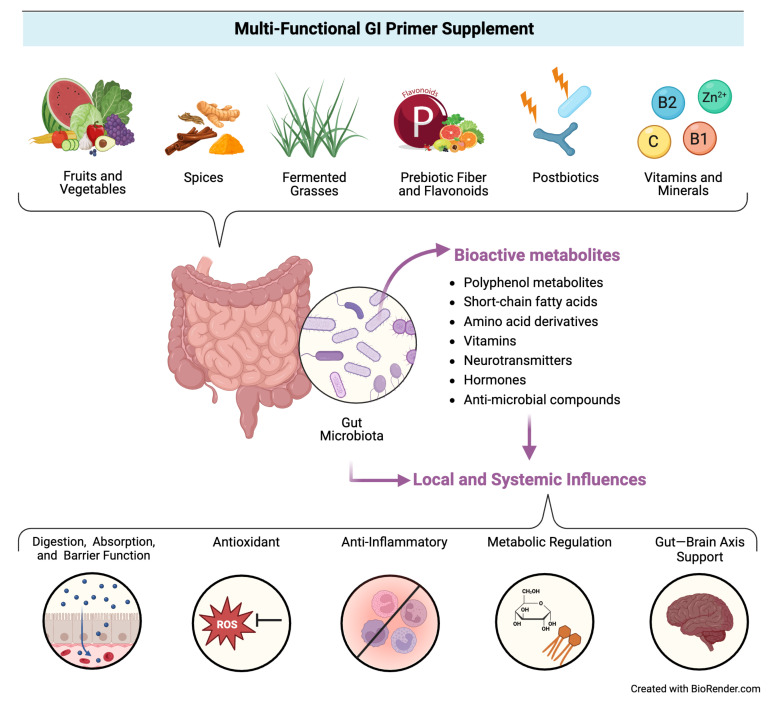
Conceptual model for the proposed activity of the multi-functional GI primer supplement.

Accordingly, there is an emerging hypothesis that “priming” the gut through exposure to specific dietary components (e.g., phytochemicals, nutrients, prebiotics, probiotics, postbiotics, etc.) may help build microbial and enteric resilience by providing beneficial substrates for microorganisms and host cells to utilize in support of key functions related to gut health (e.g., immunoregulation, nutrient metabolism, intestinal barrier integrity, response to pathogens, etc.) [14], which in turn could mitigate disruptions to mucosal health and subsequent gastrointestinal symptoms, ultimately impacting local and systemic health. Figure 1 illustrates this conceptual model.

To probe the hypothesis, we designed a multi-functional GI primer supplement. The supplement contains a blend of functional ingredients recognized for supporting specific physiological activities that may prime the gut to promote gut microbial and enteric resilience. This blend includes fermented grasses, fruit and vegetable concentrate powders, spices, prebiotics (e.g., fiber, flavonoids), postbiotics, digestive enzymes, and other nutrients. Phytochemicals (e.g., polyphenols, nutrients) present in the plant-based components [15,16], in addition to pre- and postbiotics [17,18], have been linked to regulating immune responses, antioxidant, and cardiometabolic activities, in addition to altering gut microbial activity and promoting enhanced intestinal barrier function. In summary, these functional components may promote not only digestive and mucosal health but systemic health as well.

Thus, the primary objective of this single arm human clinical trial is to gather preliminary data examining the impact of the GI primer supplement on digestive health outcomes (e.g., self-reported GI symptoms and stool consistency). The secondary objectives of this study are to assess changes in participants’ quality of life (e.g., vitality/energy and general health) and reasons for satiety. This study provides key insight into the potential experience consumers will have after taking GI primer supplement over 14 days, in addition to preliminary clinical evidence regarding health outcomes associated with this product and its potential for use as a gut priming agent. 

## 2. Materials and Methods

### 2.1. Participants

Participants were generally healthy males and females aged 18–65 with a range of mild digestive issues. A total of 70 participants were recruited for this study, all of whom provided their informed consent to participate, with 68 participants (62 females and 6 males) continuing to the end of the study. The average age of participants was 42.57 (SD 10.37) with 18 participants (26.5%) in 18–35 age group, 28 participants (41%) in 36–49 age group, and 22 participants (32.4%) in 50–65 age group. The two dropouts occurred for the following reasons: one participant became unresponsive to follow-up and one participant had a family emergency.

All participants satisfied the following inclusion and exclusion criteria. *Inclusion criteria:* aged 18–65; history of self-reported mild digestive issues (e.g., occasional bloating, stomach discomfort, occasional constipation, or occasional diarrhea); no known allergies to the ingredients listed in the product; no history of uncontrolled chronic health conditions; and willingness to comply with study requirements. *Exclusion criteria*: history of pre-existing chronic conditions that would prevent participants from adhering to the protocol, including oncological and psychiatric disorders; history of any severe allergies that require an Epi-Pen; women who were pregnant, breastfeeding, or trying to conceive; history of diagnosed digestive disorders (e.g., Irritable Bowel Syndrome (IBS), Irritable Bowel Disease (IBD), Crohn’s disease) or gastrointestinal tract surgeries; history of invasive medical procedure within the last three weeks or planning to undergo an invasive medical procedure during the study period; history of substance abuse; anyone currently participating or planning to participate in a research study; regular consumption of probiotics, fiber, or prebiotic supplements within 3 weeks of the study; regular intake of medications that may interfere with the study or study product, including laxatives, sedatives, beta-blockers, anti-acids, etc.

### 2.2. Study Design Overview

This study was designed as a single-arm open-label clinical trial using GI primer supplement powder by Access Business Group International LLC. (Ada, MI, USA) as the intervention. All participants consumed one scoop (about 8.5 g) of the product mixed into 8–10 oz of water every morning, 10–30 min before breakfast, for two weeks (Days 1–14). Due to the virtual nature of this trial, with no in-person screening or study visits, two weeks’ worth of study product was mailed to each participant after enrollment. A placebo was not utilized as it was not feasible to design a true placebo product that would both taste and look indistinguishable from the intervention. All participants were blinded to the sponsor name, brand name, and product name. Outcomes were self-reported by participants at various endpoints from Baseline (Day 0/pre-intervention) to Day 14, using both validated and study-specific questionnaires. The trial was registered at clinicaltrials.gov (NCT06283732).

### 2.3. Study Product

The GI primer supplement was designed as a multi-functional digestive health product, and the product used in this study is a standardized formula containing the same quantities of specified constituents as the product marketed to consumers. In general, the product contains six functional blends, including 3.15 g of the Prebiotics blend (partially hydrolyzed guar gum, apple fiber, slippery elm bark, and citrus flavonoids); 2.0 g of the Fermented Grasses blend (barley grass, oat grass, alfalfa, and wheatgrass); 17.9 mg of the Postbiotics (10 mg of heat-treated *Lactobacillus plantarum* L-137 microbial cells and 2.5 billion cells of heat-treated *Bifidobacterium longum* CECT 7347); 100 mg of the Digestive Enzymes blend, containing protease 4.5 5000 HUT, amylase 2750 DU, lactase 250 ALU from *Aspergillus oryzae*, bromelain 120,000 PU from *Ananas comosus*, cellulase 125 CU from *Trichoderma longibrachiatum*, Papain 125,000 PU from *Carica papaya*, and lipase 300 FIP from *Candida rugosa*; 985 mg of Fruit and Vegetable concentrates (kale, amla, apple, artichoke, asparagus, plum, cranberry, goji, and beet); the Spices blend (ginger, fermented turmeric, fermented cinnamon, fermented cayenne, and fermented fennel seed); and select vitamins (0.40 mg thiamine, 0.45 mg riboflavin, 60 mg ascorbic acid/Vitamin C) and 2.75 mg of zinc from zinc gluconate. The total dietary fiber content per daily serving of this product, which is derived from a combination of the aforementioned ingredients, is 4.0 g (~14% DV).

### 2.4. Outcomes

The primary outcome of digestive health included the severity of gastrointestinal symptoms, assessed by daily diary entries in questionnaire format every day from Baseline to Day 14, and stool consistency measured twice by the Bristol Stool Scale at Baseline and Day 14. Secondary outcomes are as follows: Quality of life, which included measures of vitality/energy and overall general health, assessed by the Short Form 36 survey at Baseline and Day 14; and Satiety, which was assessed by the Reasons Individuals Stop Eating Questionnaire at Baseline and Day 14. Adverse events were reviewed and reported throughout the entire duration of the study.

### 2.5. Instruments

#### 2.5.1. Daily Diary Entries

The daily diary entries in questionnaire format assessed the severity of a wide range of digestive symptoms (Table S1, Supplementary Materials). These digestive symptoms encompassed gastrointestinal-specific symptoms such as thirst, stomach cramps, rumbling/stomach noise, nausea, indigestion, gas/flatulence, bloating, constipation, hunger, and acid reflux, in additional to extraintestinal symptoms that may be related to digestive disruption such as fluctuation in energy levels, brain fog/difficulty concentrating, and fatigue. Participants were also given space to qualitatively record any additional symptoms/experiences. Participants were asked to rate these symptoms on a scale of 0–3, with 0 = none/no symptoms, 1 = mild symptoms, 2 = moderate symptoms, and 3 = severe symptoms. Definitions corresponding to each category of symptom severity were provided to participants on the questionnaire (Table S1, Supplementary Materials). Although the symptoms recorded are a combination of gastrointestinal-specific and extraintestinal symptoms, for the purposes of this study, they were all referred to as digestive symptoms.

#### 2.5.2. Bristol Stool Scale

The Bristol Stool Scale (BSS; [19]) is a validated diagnostic scale assessing stool consistency. The BSS asks participants to classify their stool into one of seven categories using a visual aid: Types 1 and 2 are hard stools, respectively; Types 3 and 4 are harder and softer normal stools, respectively; Type 5 is normal with a tendency for soft stools; Types 6 and 7 are loose stools, respectively. For this study, we calculated the total number and percentage of participants who reported each category at Baseline and Day 14, in addition to the change in percentage of participants who reported each category between the two time points (i.e., percent change).

#### 2.5.3. Short Form-36

We used two of the quality-of-life subscales of the Short Form-36 (SF-36; [20]): the vitality sub-scale (i.e., energy) and the general health sub-scale. The vitality scale contains four questions related to levels of energy and fatigue, each scored on a 6-point Likert scale from 1 = “All the time” to 6 = “None of the time”. The general health subscale contains 5 questions related to participants’ perception of their overall health, each scored on a 5-point Likert scale. The first question contains a range of responses from 1 = “Excellent” to 5 = “Poor”. The remaining four questions ask participants to rate statements related to health and sickness from 1 = “Definitely true” to 5 = “Definitely false”. Scores for each subscale were calculated according to the scoring instructions in SF-36 manual and interpretation guide [21] and then normalized by converting scores to 0–100 values based on the total highest possible score as 100% and the worst possible score as 0%.

#### 2.5.4. Reasons Individuals Stop Eating Questionnaire

The Reasons Individuals Stop Eating Questionnaire (RISE-Q-15; [22,23]) is a validated questionnaire that measures different attitudes towards food and eating as reasons for satiation. Within the RISE-Q-15 there are 5 (five) constructs: decreased food appeal, physical satisfaction, planned amount, self-consciousness, and decreased priority of eating. The subscales associated with each construct are composed of three questions each for a total of 15 questions. Each question provides a statement and asks participants to rate how often each statement is a reason they stop eating during a typical dinner meal at home. Each question is scored on a 7-point Likert scale, with responses ranging from 1 = “Never” to 7 = “Always”, for 21 total possible points per subscale.

### 2.6. Statistical Analysis

All data analyzed were Likert scale scores for each outcome and associated time points, as described above. Data were checked for normality using the D’Agostino–Pearson test. Digestive symptom scores for each daily check-in were compared to Baseline using the repeated-measure one-way ANOVA or a non-parametric Friedman test, depending on the normality of the dependent variable. Scores for all other outcomes, which were assessed pre- (Baseline or Day 1) and post-intervention (Day 14), were compared using either a paired *t*-test or Wilcoxon test, based on the normality of the data. Proportions of participants who reported hard, normal, or loose stool consistencies were compared via χ^2^ test. All statistical analyses were performed in GraphPad Prism 9.0 with a significance threshold set at 0.05.

## 3. Results

### 3.1. Digestive Health Outcomes

Out of the 13 digestive symptoms self-reported daily, all 13 significantly improved from Baseline to Day 14 (*p*-values < 0.05; Table 1). These parameters were severeness of thirst, stomach cramps, rumbling/stomach noise, nausea, indigestion, gas/flatulence, bloating, constipation, fluctuation in energy levels, brain fog/difficulty concentrating, fatigue, hunger, and acid reflux. Most parameters, namely stomach cramps, rumbling/stomach noise, indigestion, gas/flatulence, bloating, constipation, fluctuation in energy levels, brain fog/difficulty concentrating, fatigue, and acid reflux, had already undergone significant changes by Day 2 (Table 1). These improvements were consistent and maintained each day throughout the study, which can be seen in Table A1 (Appendix A). These data indicate that participants’ perceived digestive health improved within 1–2 days and these changes were maintained over time.

The changes in stool consistency, which was self-reported via the BSS at Baseline and Day 14 are outlined in Table 2. We observed the percentage of participants experiencing normal stool consistencies (Types 3, 4, and 5) to increase by 41% from Baseline to Day 14, whereas the percentage of participants experiencing soft stools (Types 6 and 7) and hard stools (Types 1 and 2) decreased by 53.3% and 33.3%, respectively. The changes in proportions of hard, normal and soft stool categories were statistically significant with *p* = 0.027 (χ^2^ test). This suggests a general improvement in the consistency of bowels after consuming the GI primer supplement for 14 days.

### 3.2. GI Primer Supplement Improves Self-Reported Vitality/Energy Levels and General Health

Participants self-reported their vitality/energy and general health at Baseline and Day 14. There was a significant increase (*p*-value < 0.0001) in average vitality/energy scores from Baseline to Day 14 (Table 3), with the average score increasing by 40.39%. There was also a significant increase (*p* = 0.035) in average general health scores from Baseline to Day 14 (Table 3), with the average score increasing by 8.07%. This indicates an improvement in participants’ perceived energy levels (e.g., more energy and less fatigue) and general health with once-daily use of the product.

### 3.3. Participant Attitudes towards Food and Eating Changed after Daily Use of GI Primer Supplement

Five factors related to food and eating were assessed as being reasons for satiety. Out of these five, average scores for physical satisfaction (*p*-value = 0.0003), planned amount (*p*-value = 0.0001), and decreased priority of eating (*p*-value < 0.0001) significantly increased from Baseline to Day 14 (Table 3). These increases in scores correspond to participants reporting that they more frequently feel satiated due to eating their planned amount, feeling physically satisfied with the amount they ate, and eating no longer feeling like a priority.

Conversely, average scores for both self-consciousness and decreased food appeal significantly decreased (*p*-values < 0.0001) from Baseline to Day 14 (Table 3). For self-consciousness, a decrease in scores indicates that participants less frequently experienced self-consciousness around food and eating by Day 14, which could be interpreted as an improvement in that parameter. For decreased food appeal, a decrease in scores indicates that participants less frequently felt as if the food was no longer appealing, pleasant, or of interest by Day 14.

These data indicate that after daily use of the GI primer supplement, participants’ attitudes toward food and eating generally improved, with participants more frequently reporting attitudes related to physical or interoceptive awareness-related satiety as opposed to attitudes related to sensory or motivation-specific satiety, although the actual energy intake was not considered. 

No serious adverse events were reported during the study.

## 4. Discussion

Under the hypothesis that a multi-functional digestive health product could act as a gut-priming agent to support digestive health and these changes could translate to improved systemic health, we investigated participants’ reported changes in functional GI symptoms, stool consistency, energy levels, general health, and attitudes toward food and eating while taking a unique supplement. By Day 14, participants reported improvements in most of the parameters evaluated. Moreover, most digestive symptoms improved by Day 2, a change that was maintained until the study’s end. This supplement was also generally well received by participants, with most reporting that they liked the product.

Functional GI symptoms have been associated with a number of different gut health factors, including but not limited to gut mucosal inflammation [24,25], intestinal barrier dysfunction [26,27,28], disruptions in digestive processes [29,30], and alterations in the gut microbiome [31], the latter of which may also influence the previous factors. Accordingly, there are several interconnected mechanisms potentially underlying changes in digestive symptoms and the other study outcomes in response to the intervention, many of which are likely mediated by activities of the gut microbiome.

### 4.1. Proposed Mechanisms Underlying Changes in Digestive Outcomes

The GI primer supplement is rich in different sources of soluble and insoluble fiber (e.g., apple fiber, slippery elm bark, fruit and vegetable concentrates, fermented greens, and partially hydrolyzed guar gum). Previously, dietary fiber interventions were effective in reducing a range of functional GI symptoms in IBS [32], in addition to improving bowel movement regularity and reduced constipation-type consistency in adults and children [33,34,35]. Evidence suggests that the consumption of dietary fiber sources promotes an increased abundance of beneficial bacterial taxa (e.g., *Bifidobacteria* and *Lactobacillus*) and a reduced abundance of taxa associated with inflammation [36,37,38,39]. Moreover, gut microbial fiber fermentation leads to the production of beneficial bioactive metabolites such as butyrate and other short-chain fatty acids (SCFAs) [35,40,41].

SCFAs are potent signaling molecules that regulate local and systemic inflammatory activities, ultimately dampening immune responses. SCFAs inhibit key inflammatory signaling pathways (e.g., nuclear factor-kB) and histone deacetylase activity to regulate inflammatory gene expression, the transmigration of immune cells into tissues, and the activation and proliferation of T cells [42]. Not only do SCFAs support intestinal barrier integrity by enhancing the anti-inflammatory tone of the intestinal mucosa but SCFA signaling to enterocytes also upregulates the expression of tight junctions and other structural proteins that regulate barrier permeability [43]. Additionally, butyrate is considered a key nutrient determining the metabolic activity and growth of colonocytes. Butyrate is the preferred energy source of colonic epithelial, even when competing substrates such as glucose and glutamine are available [44], and may function as a major protective factor against colonic disorders.

While dietary fiber is a direct substrate for SCFA production, other diet-derived compounds can regulate microbial activities and, thus, SCFA production and intestinal barrier health as well. Postbiotics present in the GI primer supplement, such as heat-treated *L. plantarum* L-137 [45,46,47,48] and heat-treated *B. longum* CECT 7347 [49], were also linked to increased abundance of SCFA-producing bacteria and butyrate production and/or reduced intestinal permeability by acting as signaling molecules to microbiota and enterocytes. These proposed anti-inflammatory and intestinal barrier-supporting mechanisms may contribute to our own observations and those of previous studies in which postbiotics such as heat-treated *B. longum* CECT 7347 reduced severity of IBS-D GI symptoms in adults, including abdominal pain, GI discomfort, diarrhea-like stool consistency, and IBS-related quality of life [50]. Prebiotic citrus flavonoids [51] and zinc [52,53,54] have also displayed similar SCFA-promoting bioactivities.

Additionally, many of the plant-based dietary components found in the GI primer supplement—such as fruits and vegetable concentrates [55], fermented greens [56], and spices [57]—are reservoirs of bioactive phytochemicals and nutrients. Dietary polyphenols, for example, are a diverse group of phytochemicals widely associated with antioxidant activities, in addition to supporting healthy gut microbial community structure and function, bioactive microbial metabolite production (e.g., SCFAs, bile acids, polyphenol metabolites), and intestinal barrier integrity through multiple mechanisms [58]. These mechanisms perhaps underlie observations in which polyphenol-rich interventions improved GI symptoms, such as bowel movement consistency and frequency, abdominal pain and discomfort, bloating, and reflux [59,60,61], and observations in which phytochemical-rich interventions (e.g., ginger) improved digestive function, gastric emptying, gastric motility, and nausea [62,63,64]. Moreover, mounting evidence suggests that the metabolism of polyphenols and other phytochemicals by gut microbiota and the resultant microbial metabolites are largely responsible for their bioactivities [58].

Finally, the use of digestive enzymes (e.g., protease, lipase, amylase, lactase) improved digestion [65,66,67] and GI symptoms, such as flatulence, abdominal distension, bloating, epigastric burning, abdominal pain, and stool frequency and consistency, in different contexts [68,69,70,71,72,73]; in vivo studies also indicate that *Aspergillus*-derived digestive enzymes, which is the microbial source of the protease present in GI primer supplement, may have prebiotic-like effects and promote the growth of beneficial bacteria such as *Lactobacillus* and *Bifidobacterium* [74,75].

In summary, the interaction between the aforementioned dietary components, gut microbiota, and intestinal mucosa may promote microbial and enteric resilience, thus priming the gut to support digestive health to reduce gastrointestinal-specific and associated extraintestinal symptoms.

### 4.2. Proposed Mechanisms Underlying Changes in Energy/Vitality

In this study, we also observed that two self-reported quality-of-life measures, vitality/energy, and general health, were improved by Day 14. Several vitamins found in the GI primer supplement, such as B1 [76], B2 [77], and C [78,79], play a role in energy production and energy-yielding metabolism, which could potentially impact participants’ perceived energy/fatigue. Indeed, previous clinical investigations into the relationships between these vitamins and patient-reported outcomes also observed improved quality of life, reduced fatigue/increased energy, and the improved well-being of participants in different contexts [80,81,82,83,84,85,86,87]. Several components present in the GI primer supplement may also affect perceived energy levels via enhanced nutrient absorption. Nutrient deficiencies and/or supplementation in nutrient-deficient contexts were associated with self-reported energy and fatigue [88,89,90]. Moreover, nutrient deficiencies and fatigue have been observed in gastrointestinal disorders [91,92,93,94,95]. The use of exogenous digestive enzymes not only improves the digestibility of major macromolecules [65,66,67] but enhances the bioavailability of digestive end products [68,69,70]. Soluble fiber fermentation by gut microbiota can also influence nutrient uptake, as higher levels of butyrate (SCFA) are associated with lower luminal pH values in the intestinal tract, which can increase the uptake and absorption of key electrolytes (e.g., sodium and calcium) and other nutrients/minerals [35,96]. Furthermore, the fermentation of plant ingredients, including the fermented grasses and spices in the GI primer supplement, increases nutrient availability and absorption by breaking down complex cellulose matrices, making any nutrients (fiber, minerals, etc.) or phytochemicals entrapped in these complex matrices more accessible for absorption [56,97]. It is important to note these proposed mechanisms of improved vitality/energy may also be related to the improvement in fatigue reported on the digestive symptom questionnaire.

### 4.3. Proposed Mechanisms Underlying Changes in Reasons for Satiety

Finally, participants in this study also reported changes in reasons for satiety from Baseline to Day 14, reflecting changes in specific attitudes toward food and eating. Scores for physical satisfaction, eating a planned amount, and decreased priority of eating increased, while those for self-consciousness and decreased food appeal decreased. These findings suggest participants’ reasons for stopping to eat a meal shifted away from constructs related to sensory or motivation-specific satiety and towards constructs more related to physical or interoceptive awareness-related satiety. Total dietary fiber and individual fiber sources (e.g., partially hydrolyzed guar gum and apple fiber) were previously shown to increase satiety and reduce post-meal energy intake [35,98,99], with some specific sources (e.g., partially hydrolyzed guar gum) showing both immediate and long-term effects on satiety, hunger, appetite, and desire to eat [100,101]. Additionally, fiber [35,102,103], grasses (e.g., cereal and barley; [104]), and ginger [105] interventions have attenuated postprandial glucose and/or insulin responses, although evidence on the relationship between subjective satiety and postprandial glucose and/or insulin responses is conflicting; some studies report these factors to be related and/or co-occurring, while some studies report otherwise [106,107,108,109,110]. Moreover, the regulation of postprandial glucose response may also underlie the improvement in brain fog/difficultly concentrating scores, as self-reported in the digestive symptoms questionnaire. Indeed, postprandial glucose response has been linked to cognitive function in various contexts [111].

Another physiological mechanism linking dietary factors to satiety is gut–brain axis communication. The gut–brain axis, or the bi-directional line of communication between the central nervous system and the gut, can impact satiety and appetite and is partly regulated by the gut microbiome [112]. Not only do some microbial metabolites influence the production of hormones and neurotransmitters but some microbial metabolites are neurotransmitters and hormones [113]. Accordingly, maintaining a healthy gut microbiome via interaction with dietary factors is crucial to gut–brain axis signaling. SCFAs that result from microbial fiber fermentation, for example, regulate the production of gut peptides (e.g., peptide YY; PYY and glucagon-like peptide; GLP) that affect neuronal signaling in areas of the brain related to appetite and satiety [114]. Clinical investigations corroborate the role of dietary fiber in PYY and GLP production, noting the increased production of these peptides in response to various fiber interventions [108,110].

### 4.4. Strengths and Limitations

This study has both strengths and limitations. A notable strength of this study is its contribution to the literature in providing preliminary evidence supporting the use of multi-functional dietary products for priming the gut to promote GI health. Additionally, by using validated participant-reported outcomes, the results of this study directly reflect the participant’s experience with this product, both in terms of perceived health benefits and product experience. Moreover, the use of the RISE-Q-15 in this context provides unique insight into participants’ attitudes towards food and eating that may contribute to their feeling of satiety. While it was not feasible to design a true placebo that would be indistinguishable in look and taste from the study product, we acknowledge that the absence of a comparator group is a limitation of the study design. Another limitation of this study is the 14-day duration, which makes it impossible to assess long-term effects of the supplement and the stability of the observed improvements. Future studies evaluating this product would benefit from alternative trial designs, e.g., cross-over trials, or wait list controls to assess typical variability in the measures used, in addition to an increased duration that is able to assesses the outcomes over a longer period of time. Furthermore, fecal sample collection and gut microbiome analysis would provide more mechanistic insight into the gut priming and microbial resilience hypothesis.

## 5. Conclusions

Cumulatively, this two-week study provides key insight into participant perceptions of the GI primer supplement on digestive health, energy/vitality, general health, and satiety outcomes during the duration of the study. Results suggest that the daily consumption of the GI primer supplement may be useful in supporting these outcomes, with digestive symptoms beginning to improve within 1–2 days of consumption and lasting through day 14. The GI primer supplement contains a variety of multi-functional food components, each with previously indicated gut mucosal and systemic bioactivities. This study provides preliminary evidence for the use of such multi-functional products in supporting digestive health and alleviating functional GI symptoms over the course of two weeks. The results of this preliminary study just future investigations into the long-term effects of this GI primer supplement, including additional biomarker and mechanistic (e.g., microbiota) outcomes.

## Figures and Tables

**Table 1:** Average scores of reported digestive symptoms at Baseline and select time points.

	Baseline	Day 1			Day 2			Day 7			Day 14		
	M (SD)	M (SD)	% Change	*p*-Value	M (SD)	% Change	*p*-Value	M (SD)	% Change	*p*-Value	M (SD)	% Change	*p*-Value
Thirst	0.971 (0.646)	0.956 (0.700)	−1.52	>0.9999	0.794(0.724)	−18.2	>0.9999	0.706 (0.648)	−27.3	0.55	0.559 (−0.678)	−42.4	**0.0093**
Stomach cramps	1.132 (0.790)	0.735 (0.840)	−35.1	0.0675	0.632 (0.771)	−44.2	**0.0049**	0.456 (0.700)	−59.7	<0.0001	0.2647 (0.589)	−76.6	**<0.0001**
Rumbling or Stomach Noise	1.309 (0.696)	0.927 (0.759)	−29.2	0.1388	0.765 (0.735)	−41.6	**0.0041**	0.471 (0.634)	−64.	<0.0001	0.382 (0.599)	−70.8	**<0.0001**
Nausea	0.721 (0.709)	0.588 (0.833)	−18.4	>0.9999	0.397 (0.756)	−44.9	0.0617	0.338 (0.637)	−53.1	0.0274	0.221 (0.514)	−69.4	**0.0004**
Indigestion	1.250 (0.780)	0.927 (0.886)	−25.9	0.3151	0.632 (0.809)	−49.4	**0.0001**	0.485 (0.68)	−61.2	<0.0001	0.235 (0.522)	−81.2	**<0.0001**
Gas or Flatulence	1.50 (0.635)	1.118 (0.820)	−25.5	**0.013**	1.059 (0.751)	−29.4	**0.0011**	0.868 (0.751)	−42.2	<0.0001	0.662 (0.745)	−55.9	**<0.0001**
Bloating	1.574 (0.740)	1.147 (0.885)	−27.1	0.1173	0.941 (0.862)	−40.2	**0.0014**	0.75 (0.817)	−52.3	<0.0001	0.574 (0.676)	−63.6	**<0.0001**
Constipation	1.1765 (0.772)	0.927 (0.919)	−21.3	0.5373	0.706 (0.793)	−40.0	**0.0332**	0.309 (0.629)	−73.8	<0.0001	0.353 (0.686)	−70.0	**<0.0001**
Fluctuation in Energy Levels	1.794 (0.783)	1.177 (0.961)	−34.4	**0.0018**	1.044 (0.921)	−41.8	**<0.0001**	0.691 (0.778)	−61.5	<0.0001	0.515 (0.743)	−71.3	**<0.0001**
Brain Fog/Difficulty Concentrating	1.412 (0.918)	1.015 (0.889)	−28.1	0.1824	0.838 (0.891)	−40.6	**0.0039**	0.559 (0.761)	−60.4	<0.0001	0.397 (0.649)	−71.9	**<0.0001**
Fatigue	1.588 (0.868)	1.265 (0.857)	−20.4	**0.0187**	1.059 (0.976)	−33.3	**<0.0001**	0.735 (0.785)	−53.7	<0.0001	0.647 (0.728)	−59.3	**<0.0001**
Hunger	1.015 (0.782)	0.971 (0.732)	−4.3	0.9994	0.809 (0.718)	−20.3	0.2748	0.515 (0.68)	−49.3	0.0001	0.485 (0.635)	−52.2	**0.0002**
Acid Reflux	0.971 (0.962)	0.588 (0.868)	−39.4	0.646	0.3823 (0.6698)	−60.6	**0.0065**	0.338 (0.588)	−65.2	0.002	0.235 (0.601)	−75.8	**<0.0001**

Table 1 shown are average scores (M) of reported digestive symptoms and their standard deviations (SD), with scores ranging from 0–3 (none to worst). A decrease in the average score corresponds with an improvement in that parameter. Percent (%) change is the change from baseline values. Any *p*-value bolded and in a grey box reflects a statistically significant change (*p* < 0.05) at indicated time points compared to baseline.

**Table 2:** The number and percentage of participants reporting Bristol Stool Scale (BSS) grades at Baseline and Day 14.

	Baseline	Day 14	
BSS Grade	*N* (%)	*N* (%)	% Change Days 1–14
Grade 1	7 (10.3)	3 (4.4)	−57.1
Grade 2	14 (20.6)	11 (16.2)	−21.4
Grade 3	17 (25.0)	20 (29.4)	17.7
Grade 4	8 (11.8)	19 (27.9)	137.5
Grade 5	9 (13.2)	9 (13.2)	0
Grade 6	12 (17.6)	4 (5.9)	−66.7
Grade 7	1 (1.5)	1 (2.9)	0
Normal (Grades 3, 4, 5)	34 (50.0)	48 (70.6)	41.2
Hard stools (Grades 1, 2)	21 (30.9)	14 (20.6)	−33.3
Soft Stools (Grades 6, 7)	13 (19.1)	6 (8.8)	−53.9

Table 2 shown are the total number (N) and percentage (%) of participants who reported BSS grade (grades 1–7) corresponding with either types of hard, normal, or loose consistency stool at Baseline and Day 14. Also shown is the change in the percentage of participants who reported each stool grade from Baseline to day 14.

**Table 3:** Changes in quality-of-life measures and reasons for satiety from Baseline to Day 14.

		Baseline	Day 14		Paired *t* Test
Instrument	Score Range	M (SD)	M (SD)	% Change from Baseline	*p*-Value
SF-36					
Vitality/Energy	0–100	43.25 (15.90)	60.72 (18.04)	40.39	<0.0001
General Health	0–100	67.86 (15.49)	73.32 (14.37)	8.04	0.035
RISE-Q-15					
Decreased Food Appeal	0–21	14.68 (3.65)	8.43 (3.88)	−42.59	<0.0001
Physical Satisfaction	0–21	12.63 (2.61)	14.13 (2.48)	11.87	0.0003
Planned Amount	0–21	13.24 (3.47)	15.07 (3.1)	13.89	0.0001
Self-Consciousness	0–21	14.01 (3.53)	9.35 (3.08)	−33.26	<0.0001
Decreased Priority of Eating	0–21	12.25 (2.06)	15.82 (4.68)	29.17	<0.0001

Table 3 shown are average scores (M) for SF-36 and RISE-Q-15 subscales and their standard deviations (SD) at Baseline and Day 14. Percent (%) change is the change from baseline values.

## Data Availability

The data presented in this study are available within this article.

## Supplementary Materials

The following supporting information can be downloaded at: https://www.mdpi.com/article/10.3390/nu16183173/s1. Table S1: Daily dairy template for recording digestive symptoms.

## Appendix A

**Table A1:** Average scores of reported digestive symptoms at all time points.

		Thirst	Stomach Cramps	Rumbling/Stomach Noise	Nausea	Indigestion	Gas/Flatulence	Bloating	Constipation	Fluctuation in Energy Levels	Brain Fog/Difficulty Concentrating	Fatigue	Hunger	Acid Reflux
Baseline	M	0.9706	1.1324	1.3088	0.7206	1.25	1.5	1.5735	1.1765	1.7941	1.4118	1.5882	1.0147	0.9706
	SD	0.6458	0.7899	0.6966	0.7091	0.7799	0.6348	0.7394	0.7715	0.7834	0.9181	0.8679	0.7821	0.9615
Day 1	M	0.9559	0.7353	0.9265	0.5882	0.9265	1.1176	1.1471	0.9265	1.1765	1.0147	1.2647	0.9706	0.5882
	SD	0.7004	0.8396	0.7593	0.8328	0.8863	0.8201	0.8854	0.9194	0.9611	0.8893	0.8572	0.7324	0.8679
	% Change	−1.5152	−35.0649	−29.2135	−18.3673	−25.8824	−25.4902	−27.1028	−21.25	−34.4262	−28.125	−20.3703	−4.3478	−39.3939
Day 2	M	0.7941	0.6323	0.7647	0.3970	0.63235	1.05882	0.94118	0.70588	1.04412	0.83824	1.05882	0.80882	0.3823
	SD	0.7239	0.7708	0.7354	0.7558	0.80862	0.75077	0.86183	0.79286	0.92129	0.89126	0.97556	0.71774	0.6698
	% Change	−18.1818	−44.1558	−41.5730	−44.8979	−49.4117	−29.4117	−40.1869	−40	−41.8032	−40.625	−33.3333	−20.2898	−60.6060
Day 3	M	0.8382	0.5735	0.75	0.3971	0.5882	1.0735	1.0441	0.5588	0.8529	0.7206	1.1029	0.6912	0.4265
	SD	0.8944	0.8165	0.8944	0.4082	0.4082	0.8944	0.9832	0.8165	0.8165	0.8367	0.8165	0.8165	0.5164
	% Change	−13.6364	−49.3506	−42.6966	−44.8979	−52.9411	−28.4313	−33.6448	−52.5	−52.4590	−48.9583	−30.5555	−31.8840	−56.0606
Day 4	M	0.8088	0.4412	0.5882	0.3529	0.4853	1	0.9265	0.4412	0.8971	0.6324	0.8382	0.7059	0.3971
	SD	0.6966	0.608	0.6744	0.6173	0.68	0.7727	0.852	0.6776	0.8311	0.7899	0.7844	0.7543	0.7153
	% Change	−16.6666	−61.0389	−55.0561	−51.0204	−61.1764	−33.3333	−41.1214	−62.5	−50	−55.2083	−47.2222	−30.4347	−59.0909
Day 5	M	0.6912	0.5294	0.6176	0.3088	0.4706	0.9706	0.8382	0.4118	0.8676	0.6765	0.8971	0.6912	0.3529
	SD	0.6049	0.7012	0.713	0.6291	0.6341	0.7324	0.8215	0.6519	0.8962	0.8715	0.8489	0.7966	0.5926
	% Change	−28.787	−53.246	−52.808	−57.142	−62.3529	−35.2941	−46.7289	−65	−51.6393	−52.0833	−43.5185	−31.8840	−63.6363
Day 6	M	0.7059	0.5147	0.5	0.3676	0.4412	0.8382	0.7647	0.4706	0.8676	0.6029	0.8824	0.6618	0.3382
	SD	0.6924	0.7226	0.6579	0.6442	0.6776	0.6604	0.7354	0.6572	0.7899	0.7944	0.8381	0.7453	0.6374
	% Change	−27.2727	−54.5454	−61.7977	−48.9795	−64.7058	−44.1176	−51.4018	−60	−51.6393	−57.2916	−44.4444	−34.7826	−65.1515
Day 7	M	0.7059	0.4559	0.4706	0.3382	0.4853	0.8676	0.75	0.3088	0.6912	0.5588	0.7353	0.5147	0.3382
	SD	0.6478	0.7004	0.6341	0.6374	0.68	0.7512	0.8173	0.6291	0.7776	0.7606	0.7845	0.68	0.5887
	% Change	−27.2727	−59.7402	−64.0449	−53.0612	−61.1764	−42.1568	−52.3364	−73.75	−61.4754	−60.4166	−53.7037	−49.2753	−65.1515
Day 8	M	0.6765	0.3382	0.3971	0.25	0.3971	0.75	0.6765	0.4265	0.75	0.5588	0.8088	0.5588	0.3235
	SD	0.6566	0.6826	0.602	0.5	0.602	0.7202	0.7618	0.6979	0.7988	0.7606	0.7382	0.6776	0.5844
	% Change	−30.3030	−70.1298	−69.6629	−65.3061	−68.2352	−50	−57.0093	−63.75	−58.1967	−60.4166	−49.0740	−44.9275	−66.6666
Day 9	M	0.6324	0.4706	0.4118	0.25	0.3529	0.6618	0.5882	0.4118	0.7059	0.4559	0.7941	0.6029	0.25
	SD	0.667	0.8006	0.6286	0.529	0.5926	0.7042	0.6962	0.6519	0.8115	0.7617	0.8735	0.7153	0.5
	% Change	−34.8484	−58.4415	−68.5393	−65.3061	−71.7647	−55.8823	−62.6168	−65	−60.6557	−67.70833	−50	−40.5797	−74.2424
Day 10	M	0.6618	0.3676	0.3824	0.25	0.3382	0.7941	0.5	0.3676	0.6176	0.5147	0.7059	0.4853	0.25
	SD	0.6374	0.5961	0.6917	0.5565	0.5628	0.7641	0.6579	0.6206	0.8291	0.8374	0.882	0.68	0.5
	% Change	−31.8181	−67.5324	−70.7865	−65.3061	−72.9411	−47.0588	−68.224	−68.75	−65.5737	−63.5416	−55.5555	−52.1739	−74.2424
Day 11	M	0.6324	0.3235	0.4412	0.3529	0.3382	0.7647	0.5588	0.3824	0.6324	0.4118	0.6471	0.5294	0.3088
	SD	0.689	0.6094	0.632	0.7074	0.6374	0.7354	0.7408	0.6471	0.8447	0.7578	0.8598	0.7222	0.5797
	% Change	−34.8484	−71.4285	−66.2921	−51.0204	−72.9411	−49.0196	−64.4859	−67.5	−64.7540	−70.8333	−59.2592	−47.8260	−68.1818
Day 12	M	0.6176	0.3970	0.3970	0.25	0.2794	0.72058	0.6029	0.2647	0.6470	0.5147	0.6323	0.5	0.2205
	SD	0.7336	0.6722	0.6019	0.5827	0.5421	0.7500	0.775	0.5888	0.8060	0.7628	0.7899	0.6802	0.4839
	% Change	−36.3636	−64.9350	−69.6629	−65.3061	−77.6470	−51.9607	−61.6822	−77.5	−63.9344	−63.5416	−60.1851	−50.7246	−77.2727
Day 13	M	0.6470	0.3382	0.3088	0.2352	0.2794	0.5735	0.5588	0.3823	0.5441	0.5	0.6470	0.5441	0.2352
	SD	0.6410	0.6135	0.6048	0.5495	0.5689	0.7189	0.6776	0.6917	0.8363	0.8011	0.7873	0.6563	0.5216
	% Change	−33.3333	−70.1298	−76.4044	−67.3469	−77.6470	−61.7647	−64.4859	−67.5	−69.6721	−64.5833	−59.2592	−46.3768	−75.7575
Day 14	M	0.5588	0.2647	0.3823	0.2205	0.2352	0.6617	0.5735	0.3529	0.5147	0.3970	0.6470	0.4852	0.2352
	SD	0.6776	0.5888	0.5992	0.5138	0.5216	0.7453	0.6761	0.6859	0.7429	0.6496	0.7282	0.6346	0.6014
	% Change	−42.4242	−76.6233	−70.7865	−69.3875	−81.1764	−55.8823	−63.5514	−70	−71.3114	−71.875	−59.2592	−52.1739	−75.757

Table A1 shown are average scores (M) of reported digestive symptoms and their standard deviations (SD), with scores ranging from 0–3 (none to worst). A decrease in the average score corresponds with an improvement in that parameter. Percent (%) change is the change from baseline values.
