# Supplementary material for: Changes in Digestive Health, Satiety and Overall Well-Being after 14 Days of a Multi-Functional GI Primer Supplement
Source: Nutrients. 2024 Sep 19;16(18):3173. doi: 10.3390/nu16183173 (PMC11434699; doi:10.3390/nu16183173)
Supplement: Supplementary file 1 [file nutrients-16-03173-s001.zip › nutrients-3176115-supplementary.pdf]

Table S1. Daily dairy template for recording digestive symptoms.

<table>
<tr><td>#</td><td>Daily Diary (Every Day Baseline to Day 14)</td><td>Response Group</td></tr>
<tr><td colspan="3">How Severe Would you Rate Your:</td></tr>
<tr><td>1</td><td>Thirst</td><td rowspan="13">None = 0 no symptoms

Mild = 1 awareness of symptom, but easily tolerated

Moderate = 2 discomfort sufficient to cause interference with normal activities

Severe = 3 incapacitating, with inability to perform normal activities</td></tr>
<tr><td>2</td><td>Stomach cramps</td></tr>
<tr><td>3</td><td>Rumbling/stomach noise</td></tr>
<tr><td>4</td><td>Nausea</td></tr>
<tr><td>5</td><td>Indigestion</td></tr>
<tr><td>6</td><td>Gas/Flatulence</td></tr>
<tr><td>7</td><td>Bloating</td></tr>
<tr><td>8</td><td>Constipation</td></tr>
<tr><td>9</td><td>Fluctuation in Energy Levels</td></tr>
<tr><td>10</td><td>Brain Fog/ Difficulty to concentrate</td></tr>
<tr><td>11</td><td>Fatigue</td></tr>
<tr><td>12</td><td>Hunger</td></tr>
<tr><td>13</td><td>Acid Reflux</td></tr>
<tr><td>14</td><td>Please record any additional symptoms/experiences (1-2 sentences).</td><td></td></tr>
</table>
